# Exposure–Response Relationship between VWF/FVIII Activity and Spontaneous Bleeding Events Following Recombinant VWF Prophylaxis in Severe VWD

**DOI:** 10.1055/s-0044-1787815

**Published:** 2024-06-27

**Authors:** Frank W.G. Leebeek, Giancarlo Castaman, Jean François Marier, Gülden Özen, Indranil Bhattacharya, Jingmei Zhang, Scarlett Wang, Yi Wang

**Affiliations:** 1 Department of Hematology, Erasmus University Medical Center, Rotterdam, The Netherlands; 2 Center for Bleeding Disorders and Coagulation, Careggi University Hospital, Florence, Italy; 3 Certara Strategic Consulting, Princeton, New Jersey, United States; 4 Takeda Development Center Americas, Inc., Cambridge, Massachusetts, United States

**Keywords:** bleeding, prophylaxis, recombinant VWF, vonicog alfa, von Willebrand disease, factor VIII

## Abstract

**Background** Recombinant von Willebrand factor (rVWF, vonicog alfa, Takeda Pharmaceuticals USA) is indicated in adults diagnosed with von Willebrand disease (VWD). In this study, the exposure–response (ER) relationship between VWF activity (VWF:RCo) or factor VIII activity (FVIII:C) and spontaneous bleeding events (BEs) was evaluated in adults with severe VWD receiving rVWF prophylaxis for up to 1 year.

**Methods** This secondary analysis included 23 patients receiving rVWF prophylaxis in the open-label, phase 3 prophylaxis trial (NCT02973087). Population pharmacokinetic (PK) and PK/pharmacodynamic (PD) models were used to characterize VWF activity and endogenous FVIII:C, and PK/PD simulations were linked to spontaneous BEs to develop an ER model.

**Results** None of the five patients with VWD types 1 or 2A/B experienced spontaneous BEs. Five of 18 patients with VWD type 3 experienced ≥1 spontaneous BEs. An ER relationship was observed whereby higher VWF:RCo levels were associated with a numerically lower spontaneous BE risk ( *p* < 0.10). This relationship was independent of patients' pretrial VWF treatment. A statistically significant ER relationship was observed after accounting for relevant data (average ± standard error exposure estimate for VWF:RCo over 24 hours prior to the spontaneous BE: −0.043 ± 0.021, *p* = 0.041). The model-generated hazard ratio for a 10 IU/dL increment in the average exposure of VWF:RCo 24 hours before a spontaneous BE was 0.651 (95% confidence interval: 0.431–0.982).

**Conclusions** This ER analysis suggests a causal association between VWF:RCo and spontaneous BEs, with an increase of VWF:RCo exposure leading to a decrease in spontaneous BE risk.

## Introduction

von Willebrand disease (VWD) is the most common inherited bleeding disorder, with an estimated prevalence of 0.6 to 1.3% in the general population. 1 2 However, the proportion of patients with symptomatic VWD who require treatment is rare, with a prevalence of 23 to 113 per million population, or up to 1 in 1,000 in certain clinical settings. 3 4 VWD is caused by a deficiency or dysfunction of von Willebrand factor (VWF). 5 6 VWF is essential for primary hemostasis as it mediates platelet adhesion to the subendothelium at sites of vascular injury. VWF also influences secondary hemostasis by stabilizing factor VIII (FVIII) in the circulation. 5 6 7 Although VWD is mainly characterized by mucocutaneous bleeding, the phenotype and severity of bleeds vary between individuals and by VWD type. 8 If not adequately controlled, bleeds in patients with VWD can cause long-term complications, such as arthropathy and anemia 5 9 and lower health-related quality of life, especially in the most severely affected patients. 10 11

Recent international guidelines for the management of VWD conditionally recommend using long-term prophylaxis in patients with a history of severe and frequent bleeds. 12 Human recombinant VWF (rVWF, vonicog alfa, VONVENDI [United States]/VEYVONDI [Europe], Takeda Pharmaceuticals USA, Lexington, Massachusetts, United States) is approved for the on-demand treatment and control of bleeding events (BEs), and perioperative bleeding management in adults with VWD, as well as routine prophylaxis to reduce the frequency of BEs in adults with severe type 3 VWD receiving on-demand treatment in the United States. 13 In Europe, rVWF is approved for the prevention and treatment of hemorrhage or surgical bleeding in adults with VWD when desmopressin treatment alone is ineffective or contraindicated. 14 The approvals for prophylaxis were based on results from an international, open-label, phase 3 trial (NCT02973087) in which the efficacy and safety of rVWF prophylaxis was evaluated in adults with severe VWD. 15 rVWF prophylaxis reduced treated spontaneous BEs in patients who had previously received on-demand VWF in the past 12 months, and patients who switched from plasma-derived VWF (pdVWF) prophylaxis to rVWF prophylaxis experienced a similar reduction in spontaneous BEs requiring treatment compared to levels within the past 12 months. 15 Pharmacokinetic (PK) assessments showed VWF:ristocetin cofactor activity (VWF:RCo) maximum concentration ( *C*_max_ ) to be stable over 12 months of rVWF prophylaxis. FVIII activity (FVIII:C) trough levels increased approximately fivefold from baseline to the completion of 12 months' rVWF prophylaxis in patients who had received VWF on-demand prior to study entry. 15

In the United States, the initial dose of rVWF for routine prophylaxis in patients with severe type 3 VWD receiving on-demand treatment is 40 to 60 IU/kg body weight administered twice weekly (BIW); this can be adjusted up to 60 IU/kg BIW based on the frequency of BEs. 13 In Europe, the initial dose of rVWF for routine prophylaxis in patients with VWD is 40 to 60 IU/kg body weight administered BIW; this can be adjusted up to 80 IU/kg and/or an increased dose frequency of three times weekly based on the patient's condition and clinical response. 14 Increased understanding of the exposure–response relationship between VWF activity, endogenous FVIII:C, and BEs could help physicians individualize prophylaxis dosing regimens, thereby optimizing patient outcomes with rVWF prophylaxis. 16 17 This secondary analysis of data from the phase 3 rVWF prophylaxis study (NCT02973087) 15 evaluated the exposure–response relationship between VWF activity (VWF:RCo) or FVIII:C and treated spontaneous breakthrough BEs in adults with severe VWD receiving rVWF prophylaxis for up to 1 year. The aim of reporting these exposure–response relationship analyses is to support rVWF dosing recommendations for prophylaxis in patients with VWD.

## Methods

### Data Source

Population PK and PK/pharmacodynamic (PD) models were previously developed 18 19 using data from patients receiving intravenous rVWF for the on-demand and perioperative management of bleeding in three completed clinical studies (NCT00816660, 20 NCT01410227, 21 and NCT02283268 22 ). The models were then updated with data collected in the international, phase 3 rVWF prophylaxis study (NCT02973087) evaluating rVWF for prophylaxis and treatment of BEs, 15 resulting in data from 103 patients for modeling ( Supplementary Methods and Supplementary Table S1 in the Supplementary Material [online only]).

The exposure–response relationship was evaluated using treated spontaneous BE (hereafter referred to as spontaneous BEs) data from the phase 3 rVWF prophylaxis trial (NCT02973087). 15 This trial included 23 adults with severe VWD (VWF:RCo < 20 IU/dL) requiring VWF therapy (on-demand treatment with any VWF or prophylaxis with a pdVWF) during the year prior to enrolling in the study. In the prior on-demand patients, the recommended starting dose was 50 ± 10 VWF:RCo IU/kg BIW. In the switch group, the starting dose/dosing frequency was based on the prior pdVWF once weekly (QW) VWF dose equivalent (within ± 10%) divided into infusions one to three times per week (maximum: 80 VWF:RCo IU/kg per infusion). The detailed methodology (including patient eligibility) and results of the primary analysis of this study have previously been published. 15 All trials contributing data for this analysis were approved by the respective institutional review boards or independent ethics committees at all participating sites, and patients provided written informed consent.

### Assessment of Exposure–Response Relationship between VWF/FVIII and BEs

A longitudinal exposure–response analysis of spontaneous BEs from the phase 3 rVWF prophylaxis study 15 was performed using a repeated time-to-event (RTTE) model including a piecewise exponential additive model. 23 RTTE modeling can be used to examine the association of exposure information derived from PK/PD modeling and the likelihood of repetitive events over time (e.g., bleeding). 23 24 25 In the present study, the RTTE model included an exposure–response function in which the effect of VWF:RCo or FVIII:C on BEs was tested using linear models as part of the exposure–response model. The decision to use linear models was made following a standard model discrimination process (Akaike information criterion, objective function value, and graphical representations of goodness of fit; data not shown). In addition, a covariate test was included to account for the effect of prior therapy (VWF on-demand or pdVWF/FVIII prophylaxis).

Longitudinal VWF:RCo and FVIII:C levels for rVWF and pdVWF/FVIII were simulated using the population PK and PK/PD models along with individual patient information (described in the Supplementary Methods ), and applied as the input into the exposure–response model. Based on the population PK/PD model, the best model for the exposure–response relationship was selected from three potential models ( Supplementary Methods ). The impact of the dosing regimens (BIW or QW) on the exposure–response model for rVWF and pdVWF/FVIII was investigated based on the population PK and PK/PD model simulations. Hazard ratios (HRs) for the probability of bleeding were generated as a function of median VWF activity at steady state for patients with type 3 VWD.

## Results

### Study Population

This secondary analysis included all 23 patients receiving rVWF prophylaxis in the previously published phase 3 rVWF prophylaxis study (NCT02973087; Table 1 ). 15 Overall, the mean (standard deviation) age of patients was 40.6 (19.3) years, approximately half (52.2%) were male, and the majority had type 3 VWD (78.3%).

**Table 1:** Patient baseline characteristics (rVWF prophylaxis phase 3 study, full analysis set)

Characteristic	Prior on-demand group a ( *n* = 13)	Prior pdVWF/FVIII prophylaxis (switch) b ( *n* = 10)
Age, y		
Mean (SD)	38.0 (17.6)	43.9 (21.8)
Median (range)	30.0 (20–67)	34.0 (18–77)
Sex, *n* (%)		
Male	5 (38.5)	7 (70.0)
Female	8 (61.5)	3 (30.0)
Body mass index, kg/m ^2^		
Mean (SD)	23.3 (3.1)	23.3 (3.5)
Median (range)	23.6 (17.8–29.3)	23.7 (17.7–28.6)
VWD type, *n* (%)		
Type 1	2 (15.4)	1 (10.0)
Type 2A	0 (0)	1 (10.0)
Type 2B	1 (7.7)	0 (0)
Type 3	10 (76.9)	8 (80.0)
Mean (SD) PK/PD values, IU/dL	Baseline	Prior to study initiation
VWF:RCo	5.6 (10.7)	0.8 (2.6)
VWF:Ag	8.5 (15.3)	5.3 (8.9)
VWF:CB	8.2 (14.1)	3.5 (5.8)
FVIII:C	25.9 (40.6)	10.3 (12.5)

Abbreviations: FVIII, factor VIII; FVIII:C, factor VIII activity; PD, pharmacodynamics; pdVWF, plasma-derived von Willebrand factor; PK, pharmacokinetics; rVWF, recombinant von Willebrand factor; SD, standard deviation; VWD, von Willebrand disease; VWF, von Willebrand factor; VWF:Ag, von Willebrand factor:antigen; VWF:CB, von Willebrand factor:collagen binding activity; VWF:RCo, von Willebrand factor:ristocetin cofactor activity.

Source: Reproduced from Table 1 in Leebeek FWG, Peyvandi F, Escobar M, et al. Recombinant von Willebrand factor prophylaxis in patients with severe von Willebrand disease: phase 3 study results. Blood 2022;140(2):89–98. Copyright 2022 American Society of Hematology, with permission from Elsevier. 15

a Patients who were treated on-demand with any VWF product during the 12-month period prior to enrolling into this study.

b Patients who were treated prophylactically with a pdVWF/FVIII concentrate for ≥12 months prior to enrolling into this study.

### Reported On-Study Bleeding Events

**Fig. 1:**
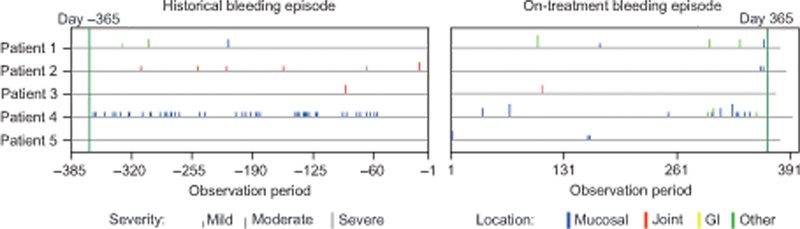
Historical and on-study treated spontaneous BEs for the five patients contributing data for the exposure–response analysis. BE, bleeding event. (Reproduced from Supplementary Fig. S1 in Leebeek FWG, Peyvandi F, Escobar M, et al. Recombinant von Willebrand factor prophylaxis in patients with severe von Willebrand disease: phase 3 study results. Blood 2022;140(2):89–98. Copyright 2022 American Society of Hematology, with permission from Elsevier. 15 )

During the study, none of the five patients with VWD type 1 or 2A/B experienced spontaneous BEs. In 18 patients with VWD type 3, there were no apparent differences in rVWF dosing between patients who had bleeding and those who did not ( Supplementary Table S2 ). Five of the 18 patients with VWD type 3 experienced ≥1 spontaneous BEs ( Table 2 ). Historical and on-study spontaneous BEs for these patients are shown in Fig. 1 . Data derived from these patients were used for the exposure–response analysis. Of these, four patients experienced multiple spontaneous BEs and one patient experienced a single spontaneous BE. The majority of spontaneous BEs were mild (14/27) or moderate (10/27); three spontaneous BEs were severe. Nineteen spontaneous BEs were of mucosal origin (nose, *n* = 14; gum, *n* = 4; mouth, *n* = 1), one was of joint origin, and seven were classified as other or missing.

**Table 2:** Treated sBEs reported during the rVWF prophylaxis phase 3 study

Patient	Treatment 12 months prior to study entry	Days on study	No. treated sBEs while on study	Study days with treated sBE occurrence
1	VWF on-demand	494	6	101, 172, 298, 333, 361, 375
2	VWF on-demand	476	3	375, 360, 365
3	pdVWF/FVIII prophylaxis	373	1	106
4	pdVWF/FVIII prophylaxis	396	13	38, 69, 251, 296, 301, 303, 311, 324, 329, 331, 338, 344, 352
5	pdVWF/FVIII prophylaxis	498	4	1, 3, 158, 161

Abbreviations: pdVWF/FVIII, plasma-derived von Willebrand factor/factor VIII; rVWF, recombinant von Willebrand factor; sBE, spontaneous bleeding event; VWF, von Willebrand factor.

### RTTE Model

Based on the population PK/PD model, the RTTE model with a linear exposure–response function linking the average VWF:RCo and FVIII:C levels over the 24 hours prior to the spontaneous BE was derived by taking into account actual dosing and information on patient characteristics in each patient. The *C*_ave_ values of VWF:RCo and FVIII:C levels 24 hours prior to spontaneous BE onset were generally lower than those observed on days without bleeding, with the exception of Patient 2 who experienced three spontaneous BEs and had 11 missing infusion records (i.e., data not captured in the database) ( Table 3 ). Patient 2 received 5 weekly doses of rVWF following the third BE, which is sufficient to bring the VWF:RCo level to a steady state. Exposure–response analyses for VWF:RCo and FVIII:C exposure metrics were performed with and without Patient 2 as the multiple missing infusion records for this patient could affect the reliability of the VWF:RCo and FVIII:C levels 24 hours prior to the BEs.

**Table 3:** Average VWF and FVIII activity associated with the treated sBEs (descriptive statistics)

	Patient 1		Patient 2 a		Patient 3		Patient 4		Patient 5	
	Days without treated sBE ( *n* = 488)	Days with treated sBE ( *n* = 6)	Days without treated sBE ( *n* = 473)	Days with treated sBE ( *n* = 3)	Days without treated sBE ( *n* = 337)	Days with treated sBE (n = 1)	Days without treated sBE ( *n* = 383)	Days with treated sBE ( *n* = 13)	Days without treated sBE ( *n* = 492)	Days with treated sBE ( *n* = 4)
VWF:RCo *C*_ave_ 24 hours prior to dosing (IU/dL)										
Mean (%CV)	14.7 (76.0)	2.95 (59.6)	13.1 (64.5)	16.3 (44.9)	7.26 (75.2)	2.64 (NA)	18.0 (61.1)	14.5 (68.4)	21.2 (63.6)	26.9 (83.5)
Median (min–max)	14.4 (0.500–60.5)	2.04 (1.55–5.32)	16.9 (0.500–42.9)	17.9 (8.29–22.6)	6.58 (0.500–23.0)	2.64 (2.64–2.64)	18.6 (0.500–69.2)	18.2 (2.02–27.4)	19.5 (0.500–60.4)	27.6 (0.500–52.2)
FVIII *C*_ave_ 24 hours prior to dosing (IU/dL)										
Mean (%CV)	108 (38.5)	83.8 (55.8)	36.7 (36.2)	50.5 (23.1)	32.9 (36.2)	26.3 (NA)	78.6 (19.7)	77.7 (16.1)	74.5 (33.3)	63.8 (60.4)
Median (min–max)	136 (5.74–159)	68.0 (38.0–142)	36.4 (10.8–72.2)	53.6 (37.6–60.3)	31.1 (14.0–60.0)	26.3 (26.3–26.3)	85.8 (11.9–88.3)	85.8 (54.2–88.1)	84.2 (6.09–97.0)	82.5 (6.09–84.2)

Abbreviations: *C*_ave_ , average level; %CV, percentage coefficient of variation; FVIII, factor VIII; max, maximum; min, minimum; NA, not applicable; rVWF, recombinant von Willebrand factor; sBE, spontaneous bleeding event; VWF, von Willebrand factor; VWF:RCo, von Willebrand factor:ristocetin cofactor activity.

a An exploratory analysis was performed after removing data from Patient 2; this patient had multiple missing infusion records ( *n* = 11) and experienced three sBEs.

### Assessment of Exposure–Response Relationship between VWF Activity and BEs

#### Analysis of all Data

A nonstatistically significant trend for the exposure–response relationship ( *p* < 0.10) was observed in the analysis of all data (including data from Patient 2), suggesting a potentially lower risk of spontaneous BE occurrence with higher exposure to VWF:RCo. An exposure estimate, which has no units and is a coefficient value linking the exposure to the probability of bleeding, was derived from the RTTE model with linear effect and used to calculate the HR. The average (±standard error) exposure estimate for VWF:RCo 24 hours prior to the spontaneous BE was −0.032 ± 0.019 ( *p* = 0.099). The HRs for simulations including all patients were nonsignificant for a 10 IU/dL and 20 IU/dL increment in the average exposure of VWF:RCo 24 hours before a spontaneous BE (HR per 10 IU/dL: 0.731, 95% confidence interval [CI]: 0.502–1.06; HR per 20 IU/dL: 0.533, 95% CI: 0.252–1.13). The effect of previous treatment on the exposure–response relationship in all patients (on-demand with a VWF or prophylaxis with pdVWF/FVIII) was not statistically significant ( *p* = 0.656).

#### Analysis with Patient 2 Excluded

A statistically significant exposure–response relationship ( *p* < 0.05) was observed, whereby a higher exposure to VWF:RCo was associated with a lower risk of spontaneous BE occurrence. The average (±standard error) exposure estimate for VWF:RCo over 24 hours prior to the spontaneous BE was −0.043 ± 0.021 ( *p* = 0.041). Simulations indicated that the HR for a 10 IU/dL increment in the average exposure of VWF:RCo 24 hours before a spontaneous BE was 0.651 (95% CI: 0.431–0.982). The HR for a 20 IU/dL increment in the average exposure of VWF:RCo 24 hours before a spontaneous BE was 0.424 (95% CI: 0.186–0.964).

**Fig. 2:**
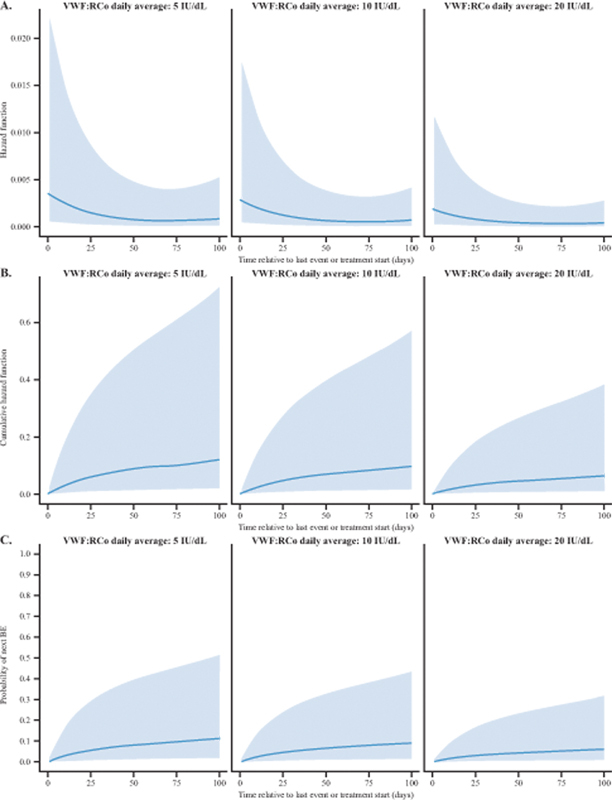
( **A** ) Hazard function, ( **B** ) cumulative hazard function, and ( **C** ) probability of next BE by daily average levels of VWF:RCo. The solid line depicts the mean values, and the shaded area represents the 95% confidence intervals. The time on the *x* -axis represents the time elapsed after each spontaneous BE; because patients can have multiple BEs, the time is reset after each BE. Hazard function represents the instantaneous potential of having a treated spontaneous BE per unit of time. The cumulative hazard function represents the total accumulated risk of experiencing the next BE that has been gained by progressing to time *t* . BE, bleeding event; VWF:RCo; von Willebrand factor:ristocetin cofactor activity.

**Fig. 3:**
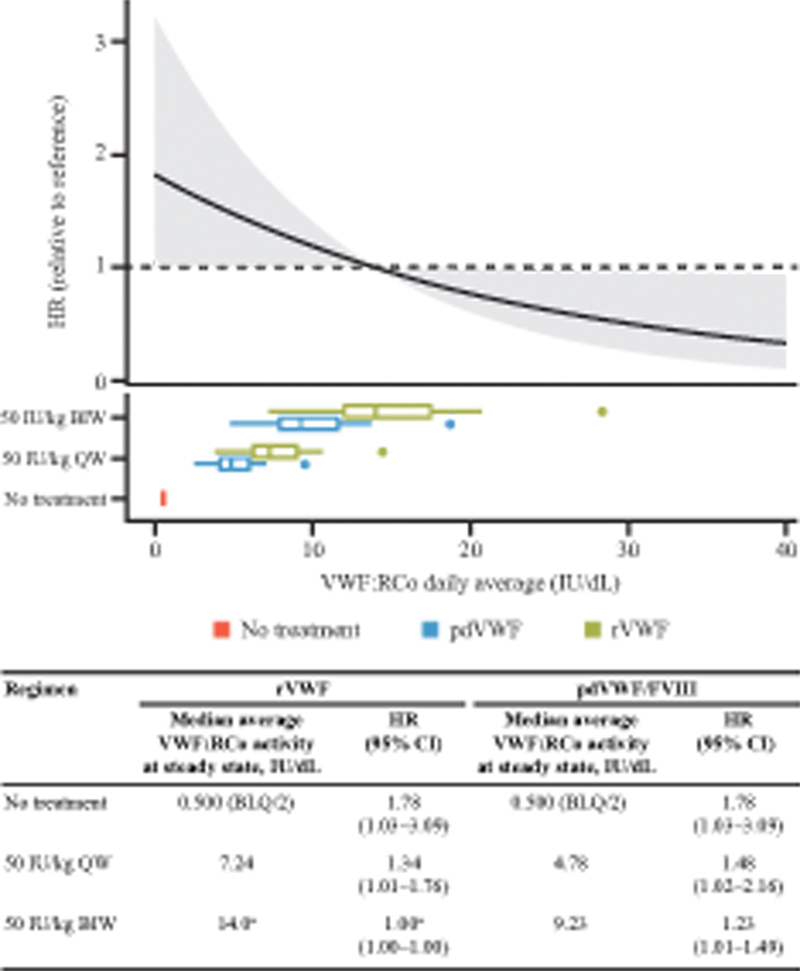
HR for the probability of bleeding for twice-weekly and once-weekly dosing of rVWF and pdVWF/FVIII products in patients with VWD type 3. BIW, twice weekly; BLQ, below limit of quantitation; CI, confidence interval; FVIII, factor VIII; HR, hazard ratio; pdVWF plasma-derived von Willebrand factor; QW, once weekly; rVWF, recombinant von Willebrand factor; VWD, von Willebrand disease; VWF:RCo; von Willebrand factor:ristocetin cofactor activity. ^a^ Reference.

The hazard function, cumulative hazard function, and probability of next BE according to VWF:RCo average levels of 5, 10, and 20 IU/dL are presented in Fig. 2 . Higher daily average VWF:RCo values were associated with lower hazards ( Fig. 2A, B ) and lower probability of next BE ( Fig. 2C ). The predicted risk of a spontaneous BE for the 50 IU VWF:RCo/kg QW regimen of pdVWF/FVIII and rVWF was 48 and 34% higher, respectively, compared with the reference regimen of rVWF 50 IU/kg BIW (HR: pdVWF/FVIII, 1.48 [95% CI: 1.02–2.16]); rVWF, 1.34 [95% CI: 1.01–1.76]). The predicted risk of bleeding with the 50 IU/kg BIW regimen of pdVWF/FVIII was 23% higher compared with the 50 IU/kg BIW regimen of rVWF (HR: 1.23 [95% CI: 1.01–1.49]) ( Fig. 3 ).

### Assessment of Exposure–Response Relationship between FVIII:C and BEs

Results derived with the RTTE model with linear effect for the average FVIII:C levels over 24 hours prior to the spontaneous BE showed that the exposure–response relationship based on FVIII:C was not statistically significant when data from Patient 2 were included in the analysis (average [±standard error] FVIII:C: −0.009 ± 0.007, *p* = 0.212). When the analysis was conducted with Patient 2 excluded, a trend was observed for the exposure–response relationship, although this was not statistically significant (average [ ± standard error] FVIII:C: −0.013 ± 0.007, *p* = 0.085). There were no significant differences in the HRs associated with median FVIII:C (all 95% CIs included 1) ( Supplementary Table S3 ). The effect of previous treatment was not statistically significant ( *p* = 0.591).

## Discussion

The findings from this secondary analysis of the phase 3 rVWF prophylaxis study 15 further clarify data from the rVWF clinical program by evaluating the PK, PK/PD, and exposure–response relationship of rVWF in patients with VWD and spontaneous BEs receiving rVWF prophylaxis for ≥1 year. Understanding this exposure–response relationship is important in achieving the long-term goal of individualizing rVWF dosing, which would enable better prediction of VWF:RCo activity, FVIII:C, and treatment effects, and potentially reduce costs. 16 17 To our knowledge, this is the first analysis in which a statistically significant exposure–response relationship has been documented in adult patients with VWD.

A RTTE model was used to examine the association of exposure information derived from the PK/PD modeling and the likelihood of bleeding. This approach accounts for the totality of exposure information over time within each patient 23 24 and is more appropriate than a count regression approach for characterizing a time-varying hazard. 26 27 Analysis of all data for exposure to VWF or endogenous FVIII:C versus spontaneous bleed occurrence indicated a relationship between VWF:RCo and spontaneous BEs requiring treatment, with higher VWF:RCo levels being associated with a lower spontaneous BE risk. This is consistent with previous studies in which patients with VWD and the lowest VWF levels had the highest bleeding scores. 28 29 The exposure–response relationship was independent of the patients' previous VWF treatment.

The impact of QW or BIW dosing regimens of rVWF and pdVWF/FVIII was also explored. In the analysis in which the patient with missing dosing information was excluded, rVWF 50 IU/kg BIW was associated with a significantly lower risk of bleeding than rVWF 50 IU/kg QW dosing or pdVWF/FVIII 50 IU/kg QW or BIW dosing. This result is interesting given the differences in these products that may influence hemostatic activity. For example, rVWF is manufactured in a genetically engineered Chinese hamster ovary cell line, which eliminates the effects of co-purifying plasma proteins, including the VWF-cleaving protease, ADAMTS13. 17 30 Therefore, rVWF contains a higher proportion of hemostatically active high-molecular-weight and ultra-large multimers compared to pdVWF. 30

Average VWF:RCo activity over 24 hours prior to bleeding onset was generally lower than that observed on days without spontaneous BEs, except for the patient with missing dosing information. This trend, however, was not observed in all patients, suggesting that other factors may play a role in the development of spontaneous BEs that require treatment. For example, treated menorrhagia episodes were considered as treated spontaneous BEs in this study and they have a different etiology than other mucosal bleeds. Patients with multiple bleeds (e.g., Patients 1 and 4) also have a greater impact on the model by contributing more bleed data.

FVIII:C levels varied between patients (e.g., Patient 1 vs. Patient 3) and Patient 3 had a FVIII:C below the recommended 40% of normal activity 13 14 with and without spontaneous BEs. This suggests that it is not possible to generalize across patients in this population regarding the level of FVIII:C that prevents spontaneous BEs. This could potentially be explained by individual differences in the association of FVIII:C level with bleeds as well as by the limited number of observations in this analysis. In contrast, the threshold for the average VWF:RCo before BEs occur may be more sensitive for associations with bleeding. In addition, the median ranges of FVIII:C and VWF:RCo activities were highly variable over time between patients with a high coefficient of variations. This may relate to inter-patient differences in the time between prior dosing and BEs and how BIW dosing was implemented.

Although the exposure–response relationship based on FVIII:C was not statistically significant for the analyses including or excluding Patient 2, the observed trend when Patient 2 was excluded is noteworthy given the small sample size, suggesting a lower risk of spontaneous BEs with increased FVIII:C. The lack of a statistically significant exposure–response relationship for FVIII:C may be due to higher baseline values of FVIII:C relative to VWF:RCo ( Table 1 ). As a result, the range of exposure values of FVIII:C available for the analysis was more limited than that of VWF:RCo, potentially preventing the identification of a statistically significant relationship. Another explanation for the lack of significance includes the mucocutaneous nature of the bleeds, given that FVIII:C may be more important for the onset of joint or muscle bleeds than mucocutaneous bleeds. 31 In support of this, joint bleeds accounted for only 1 in 27 BEs in the current study.

The difference in median average VWF:RCo between the VWF products was driven by the population PK data from the phase 1 trial (NCT00816660), which showed that, at the same doses, pdVWF/FVIII had faster clearance than rVWF, resulting in a lower exposure of VWF:RCo. 19 The triggers for spontaneous BEs are not well understood and further research is needed to explore these, which could include the use of additional clinical biomarkers.

This study had several limitations, including the small number of patients in the analysis dataset, with only 5 of 18 patients with VWD type 3 experiencing ≥1 treated spontaneous BEs. The number of covariates tested in the population PK analysis was also limited. In addition, there was a lack of VWF:RCo and FVIII:C level data collected at the onset of bleeding or during the bleeding, as well as a limitation in the types of observed bleeding. However, it is important to note that the current longitudinal analysis integrated bleeding and nonbleeding information collected over a prolonged period of treatment time, with a range of 373 to 498 days ( Table 2 ). According to the RTTE analysis, the days with no events are just as informative as days with BEs in detecting an exposure–response relationship. In addition, although Patient 2 had 11 missing dosing records over the study, the missed doses or BEs had no impact on the steady state exposure evaluated on Day 386.

## Conclusion

This exposure–response analysis suggests a causal association between VWF:RCo level and mainly mucocutaneous spontaneous BEs, with an increase of VWF:RCo exposure leading to a decrease of spontaneous BE risk. The exposure–response relationship was independent of the patients' previous treatment (VWF on demand or pdVWF prophylaxis prior to this study). Results from the present study support the recommendation of 40 to 60 IU/kg BIW prophylactic rVWF dosing for patients with VWD type 3. This relationship could be explored further when more clinical data are available and could help to individualize rVWF dosing strategies, thereby optimizing patient outcomes with prophylaxis.
