# Supplementary material for: Exposure–Response Relationship between VWF/FVIII Activity and Spontaneous Bleeding Events Following Recombinant VWF Prophylaxis in Severe VWD
Source: TH Open. 2024 Jun 27;8(2):e243–51. doi: 10.1055/s-0044-1787815 (PMC11211018; doi:10.1055/s-0044-1787815)
Supplement: Supplementary file 1 — Supplementary Material [file 10-1055-s-0044-1787815-s24040015.pdf]

Supplementary Material

Methods

The population pharmacokinetic (PK) and PK/pharmacodynamic (PD) models were developed using the data of 103 patients with $\geq$1 measurable value of VWF:RCo with von Willebrand disease (VWD) (100 patients with unique subject IDs) from four completed recombinant von Willebrand factor (rVWF; vonicog alfa, VONVENDI [United States]/VEYVONDI [Europe], Takeda Pharmaceuticals USA, Lexington, Massachusetts, United States) clinical studies (►**Supplementary Table S1**). These were: (1) a phase 1 PK, safety, and tolerability study of rVWF:recombinant factor VIII (rFVIII) and plasma-derived VWF (pdVWF)/FVIII in patients with severe VWD type 3 (NCT00816660[1]); (2) a phase 3 study of rVWF:rFVIII and rVWF in the treatment of bleeding events (BEs) in patients with severe VWD type 3 and non-type 3 VWD (NCT01410227[2]); (3) a phase 3 study of rVWF in elective surgical procedures in patients with severe VWD (NCT02283268[3]); and (4) a phase 3 rVWF prophylaxis study of rVWF as prophylaxis treatment in patients with severe VWD (NCT02973087[4]). All patients were $\geq$18 years of age and had no history or presence of VWF inhibitors or FVIII inhibitors (Bethesda assay with <0.6 Bethesda units [BU] or Bethesda assay with Nijmegen modification <0.4 BU). Analyses were performed using nonlinear mixed-effect modeling (NONMEM, version VII, level 7.4.0; ICON plc, Dublin, Ireland).

In the PK analysis, intrinsic and extrinsic sources of variability were examined to generate model parameters and evaluate predictors of heterogeneity for PK/VWF:ristocetin cofactor activity (VWF:RCo) time profiles. Intrinsic covariates included VWD type, body weight, age, sex, race, hematocrit, markers of liver function, renal function and impairment classification, and baseline factor VIII activity (FVIII:C). Extrinsic covariates included study, treatment, nominal dose, and duration of BEs.

In the PK/PD analysis, intrinsic and extrinsic sources of variability were evaluated as described for PK to generate model parameters and evaluate predictors of heterogeneity for PD/FVIII:C time profiles. The impact of covariates was assessed using a stepwise forward additive ($p = 0.01$) and backward elimination ($p = 0.001$) approach. A standard model discrimination process was used to assess the quality of model fits. Model performance was tested using a prediction-corrected visual performance check.

Exposure Metrics 1, 2, and 3 were derived for VWF:RCo PK and FVIII:C as follows:

- Exposure Metric 1 (base model): average VWF:RCo and FVIII:C levels (average level [C_{ave}]) over 24 hours before spontaneous BE onset.
- Exposure Metric 2: VWF:RCo and FVIII:C trough levels before spontaneous BE onset.
- Exposure Metric 3: average VWF:RCo and FVIII:C levels in dosing intervals before spontaneous BE onset.

Model selection was performed by comparing the goodness of fit of the base exposure–response model (Exposure Metric 1) relative to Exposure Metrics 2 and 3. The RTTE model with a linear exposure–response function linking the average levels of VWF:RCo or FVIII:C over the 24 hours before spontaneous BE onset (Exposure Metric 1) was selected as the best model, as Exposure Metrics 2 and 3 did not provide a statistically significant improvement over this base model (►**Supplementary Table S4**).

Results of Population PK and PK/PD Modeling

PK Model

Various population PK models (one- or two-compartment) including baseline values of VWF:RCo were originally constructed to describe the individual and population activity profiles of VWF:RCo PK following rVWF or pdVWF/FVIII. Of all models tested, a two-compartment model with linear elimination and baseline (endogenous) levels resulted in the best goodness of fit. A schematic of this population PK model is presented in ►**Supplementary Fig. S1**.

In covariate analyses, VWF:RCo PK was dependent on body weight, age, and VWF treatment. Parameters derived with the final model population PK model of VWF:RCo are presented in ►**Supplementary Table S5**. The population PK model included an allometric component that accounted for differences in body weight. The exponent for the effect of body weight on clearance (CL) was 0.596 (i.e., [body weight (WT)/70]$^{0.596}$). These results suggested a faster CL of VWF:RCo in individuals with a higher body weight. The exponent for the effect of body weight on central volume of distribution (V_c) was 0.598 (i.e., [WT/70]$^{0.598}$), suggesting a larger V_c of VWF:RCo in individuals with a higher body weight. Differences in CL and V_c were expected to be offset partially by the body weight-based dosing of rVWF.

The population PK model also included a component accounting for differences in age. The exponent for the effect of age on CL was −0.148 (i.e., [Age/35]$^{-0.148}$]. These results suggested a slower CL of VWF:RCo in older individuals. VWF:RCo CL was 59% faster after pdVWF administration than after rVWF administration based on the population PK analysis. Race, sex, BEs, dose (2.0–80 IU/kg), and disease type (VWD types 1/2 vs. type 3) did not have an effect on the CL or V_c of VWF:RCo. Body mass index was previously shown to have no effect on CL.[5]

 © 2024. The Author(s).

Supplementary Table S1 Baseline characteristics of PK population

Characteristic	Phase 1 safety, PK (NCT00816660;[1] $n = 30$)	Phase 3 on-demand (NCT01410227;[2] $n = 36$)	Phase 3 surgery (NCT02283268;[3] $N = 14$)	Phase 3 prophylaxis (NCT02973087;[4] $n = 23$)	Overall ($N = 100$ unique patients[a])
Median (range) age, years	34.0 (18.0–60.0)	37.0 (18.0–65.0)	39.5 (20.0–71.0)	31.0 (18.0–77.0)	36.0 (18.0–77.0)
Female/male, *n* (%)	14 (46.7)/16 (53.3)	19 (52.8)/17 (47.2)	7 (50.0)/7 (50.0)	11 (47.8)/12 (52.2)	50 (50.0)/50 (50.0)
Median (range) body weight, kg	78.9 (43.8–145)	71.2 (45.0–143)	72.8 (52.0–130)	66.7 (47.4–89.9)	72.5 (43.8–145)
Median (range) BMI, kg/m^2	27.1 (16.5–47.8)	25.0 (17.6–37.5)	25.7 (17.1–38.9)	23.6 (17.7–29.3)	24.7 (16.5–47.8)
VWD type, *n* (%)					
Type 1/2	2 (6.7)	7 (19.4)	6 (42.9)	5 (21.7)	20 (20.0)
Type 3	28 (93.3)	29 (80.6)	8 (57.1)	18 (78.3)	80 (80.0)
Product, *n* (%)					
rVWF/rFVIII	22 (73.3)	10 (27.8)	0 (0)	0 (0)	31 (31.0)
pdVWF/FVIII	8 (26.7)	0 (0)	0 (0)	0 (0)	8 (8.0)
rVWF	0 (0)	26 (72.2)	14 (100.0)	23 (100.0)	61 (61.0)
Nominal VWF:RCo dose, *n* (%)					
2 IU/kg	3 (10.0)	0 (0)	0 (0)	0 (0)	3 (3.0)
7.5 IU/kg	5 (16.7)	0 (0)	0 (0)	0 (0)	5 (5.0)
20 IU/kg	4 (13.3)	0 (0)	0 (0)	0 (0)	4 (4.0)
50 IU/kg	18 (60.0)	20 (55.6)	10 (71.4)	23 (100)[b]	69 (69.0)
80 IU/kg	0 (0)	14 (38.9)	0 (0)	0 (0)	14 (14.0)
Variable	0 (0)	2 (5.6)	4 (28.6)	0 (0)	5 (5.0)
Renal function					
Normal (≥90 mL/min)	28 (93.3)	30 (83.3)	11 (78.6)	17 (73.9)	86 (86.0)
Mild impairment (60–89 mL/min)	2 (6.7)	5 (13.9)	1 (7.1)	5 (21.7)	13 (13.0)
Moderate impairment (<60 mL/min)	0 (0)	0 (0)	0 (0)	1 (4.3)	1 (1.0)

Abbreviations: BMI, body mass index; FVIII, factor VIII; FVIII:C, factor VIII activity; PD, pharmacodynamics; pdVWF, plasma-derived von Willebrand factor; PK, pharmacokinetics; rVWF, recombinant von Willebrand factor; SD, standard deviation; VWD, von Willebrand disease; VWF:Ag, von Willebrand factor:antigen; VWF:CB, von Willebrand factor:collagen binding activity; VWF:RCo, von Willebrand factor:ristocetin cofactor activity.

[a]$N = 100$ patients with unique subject IDs from four completed rVWF clinical studies: NCT00816660, NCT01410227, NCT02283268, and NCT02973087. Two sets of PK and PD parameters were estimated in three patients who enrolled in the studies (NCT01410227 and NCT02283268) and prophylaxis study (NCT02973087) due to important differences in baseline values and/or time of enrollment across studies resulting in 103 patients with data for modeling.

[b]Prior on-demand patients received 50 ± 10 IU/kg and prior switch patients received variable doses per their prior treatment with pdVWF/FVIII products.

 © 2024. The Author(s).

Supplementary Table S2 rVWF treatment details of patients with type 3 VWD[a]

**Patients with bleeds (*n* = 5)**
n = 1, 23 IU/kg BIW, then escalated to 33 IU/kg Q3D
n = 1, 60 IU/kg TIW
n = 1, 50 IU/kg BIW, then escalated to TIW
n = 1, 50 IU/kg BIW
n = 1, 50 IU/kg BIW
**Patients without bleeds (*n* = 13)**
n = 8, 50 IU/kg BIW
n = 2, 60 IU/kg BIW
n = 1, 66 IU/kg BIW
n = 1, 40 IU/kg BIW
n = 1, 24 IU/kg BIW

Abbreviations: BIW, twice a week; *n*, number of patients; Q3D, every 3 days; rVWF, recombinant von Willebrand factor; TIW, three times weekly.
[a]Doses refer to VWF:RCo IU/kg.

Supplementary Table S3 HRs associated with FVIII activity for twice-weekly and once-weekly dosing regimens of rVWF and pdVWF

Regimen	rVWF		pdVWF	
	Median of average FVIII activity at steady state (IU/dL)	HR (95% CI)	Median of average FVIII activity at steady state (IU/dL)	HR (95% CI)
No treatment	6.94	2.12 (5.02–0.89)	6.94	2.12 (5.02–0.89)
50 IU/kg QW	39.1	1.42 (2.13–0.95)	25.8	1.68 (3.03–0.93)
50 IU/kg BIW	67.4[a]	1.00 (1.00–1.00)	44.5	1.33 (1.84–0.96)

Abbreviations: BIW, twice weekly; CI, confidence interval; FVIII, factor VIII; HR, hazard ratio; pdVWF/FVIII, plasma-derived von Willebrand factor/factor VIII; QW, once weekly; rVWF, recombinant von Willebrand factor.
[a]Reference.

Supplementary Table S4 RTTE model[a] selection for exposure–response analysis of treated sBEs

Exposure parameter	All patients		Excluding Patient 2	
	OFV	ΔOFV[b]	OFV	ΔOFV[b]
VWF:RCo exposure				
Average 24 hours prior to BE (Exposure Metric 1, Base Model)	269.110	0.000	228.127	0.000
Trough level prior to BE (Exposure Metric 2)[c]	270.397	+1.287	237.521	+9.394
Average level in dosing interval prior to BE (Exposure Metric 3)[c]	272.620	+3.510	233.483	+5.356
FVIII:C exposure				
Average 24 hours prior to BE (Exposure Metric 1, Base Model)	270.834	0.000	229.724	0.000
Trough level prior to BE (Exposure Metric 2)[c]	272.243	+1.409	232.337	+2.612
Average level in dosing interval prior BE (Exposure Metric 3)[c]	272.513	+1.679	237.978	+8.254

Abbreviations: ΔOFV, change in objective function; BE, bleeding event; FVIII:C, factor VIII activity; OFV, objective function; pdVWF/FVIII, plasma-derived von Willebrand factor/factor VIII; RTTE, repeated time-to-event; sBE, spontaneous bleeding event; VWF, von Willebrand factor; VWF:RCo, von Willebrand factor:ristocetin cofactor activity.
[a]Linear models with covariate accounting for effect of previous therapy (VWF on-demand or pdVWF/FVIII prophylaxis).
[b]For 1 degree of freedom, ΔOFV of −3.841, −6.635, and −10.828 correspond to *p*-values of <0.05, <0.01, and <0.001, respectively.
[c]Exposure–response model based on Exposure Metrics 2 and 3 did not provide a statistically significant improvement over Exposure Metric 1.

 © 2024. The Author(s).

Supplementary Table S5 Final population PK model of VWF:RCo: parameter estimates

Parameter	Estimate (RSE)	BSV (RSE)	Shrinkage
CL (dL/h)	1.96 (4.1%) × $(WT/70)^{0.596}$ × $(Age/35)^{-0.148}$ × 1.50 if prophylaxis study (NCT02973087)	27.0% (17.2%)	7.5%
V_c (dL)	37.7 (3.8%) × $(WT/70)^{0.598}$ × 1.20 if prophylaxis study (NCT02973087)	24.6% (16.0%)	5.7%
Q (dL/h)	3.55 (11.5%) × $(WT/70)^{0.596}$	NA[a]	NA[a]
V_p (dL)	15.8 (4.7%) × $(WT/70)^{0.598}$	NA[a]	NA[a]
Baseline endogenous VWF:RCo (IU/dL)	8.94 (13.4%) if VWD type 1 or type 2	56.9% (41.7%)	61.4%
	0.5 if VWD type 3 (fixed)	NA	NA
Error model	Proportional: 0.174 (2.2%)<br>Additive (IU/dL): 3.51 (3.3%)	NA	6.0%

Abbreviations: BSV, between-subject variability; CL, systemic clearance; NA, not applicable; pdVWF/FVIII, plasma-derived von Willebrand factor/factor VIII; Q, distributional clearance; RSE, relative standard error; V_c, central volume of distribution; V_p, peripheral volume of distribution; VWD, von Willebrand disease; WT, body weight.
Note: Population PK parameters are given for a typical adult patient with VWD type 3 (body weight of 70 kg, age 35 years) who received monotherapy of rVWF in study NCT00816660 (treatment of bleeding event). The CL of VWF:RCo following administration of pdVWF/FVIII was 59% faster than rVWF in the phase 3 on-demand study (NCT01410227) based on the current population PK analysis. The CL and V_c of rVWF in study NCT00816660 were 26 and 22% higher than those observed in the on-demand study (NCT01410227).
[a]BSV (RSE) and shrinkage could not be estimated for peripheral parameters (Q and V_p) due to the limited number of samples available during the terminal elimination phase.

Supplementary Table S6 Final population PK/PD parameters of FVIII: parameter estimates

Parameter	Estimate (RSE)	BSV (RSE)	Shrinkage
K_{out} (1/h)	3.21 (9.0%) × 0.566 if study NCT02973087 (prophylaxis)	30.0% (32.2%)	34.2%
V_{FVIII} (dL)	34.4 (3.7%) × $(WT/70)^{0.617}$	17.4% (35.9%)	38.8%
IC_{50} (IU/dL)	1, Fixed	NA	NA
Hill	1.95 (5.2%) if VWD type 3 × 0.589 if VWD type 1/2	29.7% (12.8%)	<1%
I_{max}	0.974 (0.3%) × 0.973 if study NCT02973087 (prophylaxis)	38.8% (26.4%)	20.0%
Baseline FVIII (IU/dL)	33.9 (4.9%) for VWD type 1/2<br>3.76 (8.6%) for VWD type 3 × 1.58 if study NCT02973087 (prophylaxis)	30.9% (12.7%)	<1%
Error model	Proportional: 0.198 (0.6%)<br>Additive: 2.56 IU/dL (2.2%)	NA	12.0%

Abbreviations: BSV, between-subject variability; FVIII, factor VIII; Hill, sigmoidal factor; IC_{50}, activity that produces 50% of the maximum effect; I_{max}, maximum inhibitory effect; K_{out}, rate constant of degradation of FVIII; NA, not applicable; RSE, relative standard error; V_{FVIII}, volume of distribution of FVIII; VWD, von Willebrand disease.
Note: The K_{out}, Hill, and baseline FVIII levels in study NCT00816660 were 87% lower, 45% lower, and 59% higher than those observed in the on-demand study (NCT01410227), respectively. In addition, the baseline FVIII in study NCT02283268 (surgery) was 18% lower. Population PD parameters are given for a typical adult patient with VWD type 3, a body weight of 70 kg in study NCT01410227 (treatment of bleeding event).

PK/PD Model

The effect of VWF:RCo on FVIII:C was modeled using an indirect response model in which the rate of degradation of FVIII (K_{out}) was inhibited by VWF:RCo (►**Supplementary Fig. S2**). In covariate analyses, the study effect of prophylaxis was statistically significant on K_{out}, maximum inhibitory effect (I_{max}), and VWD type. The effect of bleeding and disease type on K_{out}, I_{max}, and VWD type was not statistically significant.

Parameters derived with the final model population PK/PD model of FVIII:C are presented in ►**Supplementary Table S6**. rVWF maintained inhibition of FVIII:C loss for longer than pdVWF/FVIII at the same dose level.[6]

 © 2024. The Author(s).

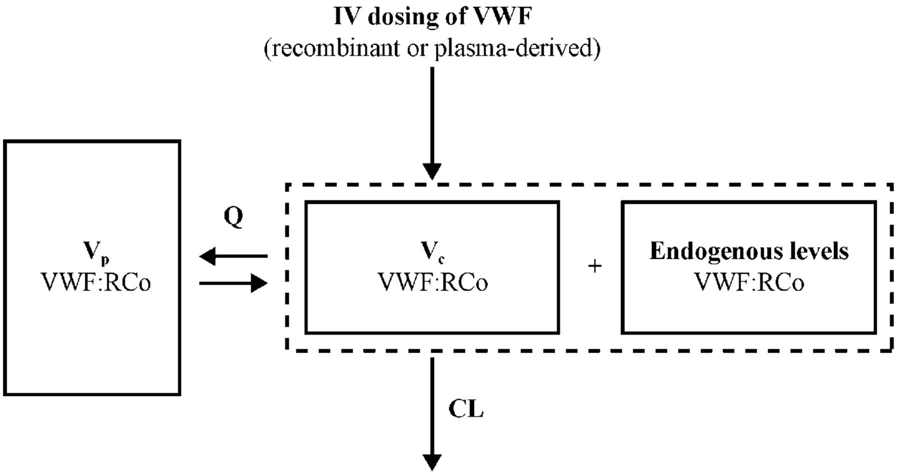

Supplementary Fig. S1 Schematic representation of base population PK model of VWF:RCo. C_{endo}, endogenous levels of VWF:RCo; CL, clearance; IV, intravenous; PK, pharmacokinetics; Q, peripheral clearance; V_c, central volume of distribution; V_p, peripheral volume of distribution; VWF, von Willebrand factor; VWF:RCo, von Willebrand factor:ristocetin cofactor activity.

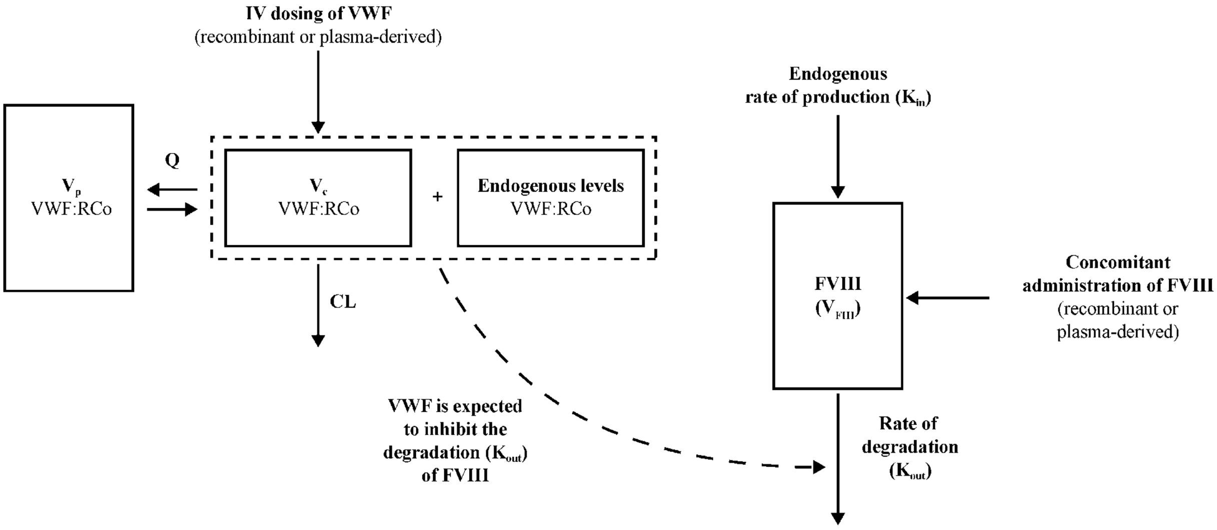

Supplementary Fig. S2 Schematic representation of PK/PD model for FVIII. CL, clearance; FVIII, factor VIII; IV, intravenous; K_{in}, endogenous rate of synthesis of FVIII; K_{out}, rate of degradation of FVIII; PD, pharmacodynamics; PK, pharmacokinetics; Q, peripheral clearance; V_c, central volume of distribution; V_{FVIII}, volume of distribution of FVIII; V_p, peripheral volume of distribution; VWF:RCo, von Willebrand factor:ristocetin cofactor activity.

References

1 Mannucci PM, Kempton C, Millar C, et al; rVWF Ad Hoc Study Group. Pharmacokinetics and safety of a novel recombinant human von Willebrand factor manufactured with a plasma-free method: a prospective clinical trial. Blood 2013;122(05):648–657

2 Gill JC, Castaman G, Windyga J, et al. Hemostatic efficacy, safety, and pharmacokinetics of a recombinant von Willebrand factor in severe von Willebrand disease. Blood 2015;126(17):2038–2046

3 Peyvandi F, Mamaev A, Wang JD, et al. Phase 3 study of recombinant von Willebrand factor in patients with severe von Willebrand disease who are undergoing elective surgery. J Thromb Haemost 2019;17(01):52–62

4 Leebeek FWG, Peyvandi F, Escobar M, et al. Recombinant von Willebrand factor prophylaxis in patients with severe von Willebrand disease: phase 3 study results. Blood 2022;140(02):89–98

5 Wang Y, Marier J, Vasilinin G, Berthoz F, Özen G. Pharmacokinetics (PK) of von Willebrand factor (VWF) in patients with von willebrand disease (VWD) after treatment with recombinant VWF or plasma-derived VWF concentrate. Clin Pharmacol Ther 2021;109 (Supplement S1):028

6 Wang Y, Marier J, Vasilinin G, Berthoz F, Özen G. Pharmacokinetic (PK) and pharmacodynamic (PD) relationship between von willebrand factor (VWF) and factor VIII (FVIII) in adult patients with von Willebrand disease (VWD). Clin Pharmacol Ther 2021;109 (Supplement S1):027

 © 2024. The Author(s).
